# Side effects and cessation of the oral contraceptive pill on TikTok: a content analysis

**DOI:** 10.3389/frph.2025.1710214

**Published:** 2025-12-10

**Authors:** Morolayo Ilori, Hannah Lee, Anisha Patel, Taylor Stanton

**Affiliations:** 1 Wayne State University School of Medicine, Detroit, MI, United States; 2 Department of Women’s Health Services, Henry Ford Health, Detroit, MI, United States

**Keywords:** oral contraceptive pill, contraception, birth control, tiktok, social media, side effects, reproductive health

## Abstract

**Objectives:**

This study aimed to assess the content and reliability of videos discussing the oral contraceptive pill (OCP) on TikTok, the popular social media platform amongst adults aged 18–24, to gauge the dialogue surrounding birth control on TikTok.

**Methods:**

We conducted a quantitative content analysis. The top 100 TikTok videos in English under each of the six hashtags related to OCPs were collected. Video content, engagement metrics (likes, comments, shares), and creator attributes were analyzed by two independent reviewers, with a third to arbitrate discrepancies.

**Results:**

307 videos were included in the final data set with an average of 134,891 likes, 1,080 comments, and 7,483 shares. Healthcare providers created 27% of videos and 85.5% of these videos were educational. The majority of videos (73%) were created by non-healthcare providers and 54.4% discussed OCPs in a negative tone. Side effects were mentioned in 79% of videos, and 64% of these videos carried a negative tone regarding OCP side effects. Discontinuing OCPs was discussed in 24% of videos, and 83% of these videos carried a negative tone.

**Conclusions:**

The most frequently discussed topic was the side effects of OCPs, with the majority framed negatively. Approximately one quarter of videos addressed discontinuing OCPs, often portraying cessation as beneficial. In the post-Roe v. Wade era, understanding how OCP experiences are portrayed on TikTok highlights the importance of physician–patient collaboration to support informed contraceptive decision-making and move beyond narratives that focus primarily on negative experiences.

## Introduction

Social media platforms offer a wealth of health information and have become a go-to destination for patient answers in the United States (U.S.). A study found that the majority of respondents, adults aged 18–48, now utilize social media platforms such as TikTok as a search engine (1). This shift in the role of social media highlights the importance of understanding what information and how this health information is presented on these platforms. Furthermore, it is well known that social networks, including friends, family members, and increasingly social media, are key sources of contraceptive information, myths, and misconceptions (2, 3). The vicarious experiences of others shared on social media can influence contraceptive decision-making and health behaviors. These online discussions occur within a rapidly evolving reproductive health landscape in the United States.

In the first half of 2024, several states enacted laws restricting access to abortion and reproductive education, while others introduced shield laws protecting abortion providers offering reproductive care from criminal consequences (4). Additionally, federal and state initiatives expanded insurance coverage for oral contraceptive supplies for up to 12 months (5, 6). These legislative shifts have heightened attention to reproductive rights and access, potentially influencing how contraception is discussed and perceived on social media platforms such as TikTok, Instagram, and YouTube.

TikTok, the popular video-sharing platform, has rapidly grown in popularity since its launch in 2016, amassing 170 million monthly users in the U.S (7). Among Americans aged 18 and 29, the majority (62%) used TikTok according to a 2023 survey conducted by Pew Research Center (8). TikTok is a unique social media platform because users engage with an algorithm-driven content feed that personalizes short-form video recommendations based on user interaction, demographics, and interests. Previous studies have explored the discussion surrounding hormonal and non-hormonal birth control methods on YouTube, Facebook, Instagram, and X (formerly known as Twitter) (9–12). It was found that birth control-related content on these social media platforms skews negatively, particularly when discussing the side effects of hormonal contraceptives (13). Although contraception-related videos on TikTok have been examined, limited research focuses exclusively on the oral contraceptive pill (OCP), the most commonly used method of reversible contraception in the U.S (14). Moreover, given the rapidly changing landscape on TikTok, it is important to produce an updated analysis of the videos that discuss OCPs.

The findings from this study will inform healthcare providers (HCPs) and the general public about the nature and accuracy of OCP-related content on TikTok. For HCPs, understanding the information patients encounter online can enhance reproductive health counselling and improve patient–provider communication. For the current and potential contraceptive users, this study offers a clearer understanding of the reliability and tone of OCP-related content encountered on social media. This descriptive study aims to assess the content and reliability of videos discussing the OCP on TikTok.

## Methods

**Figure 1:**
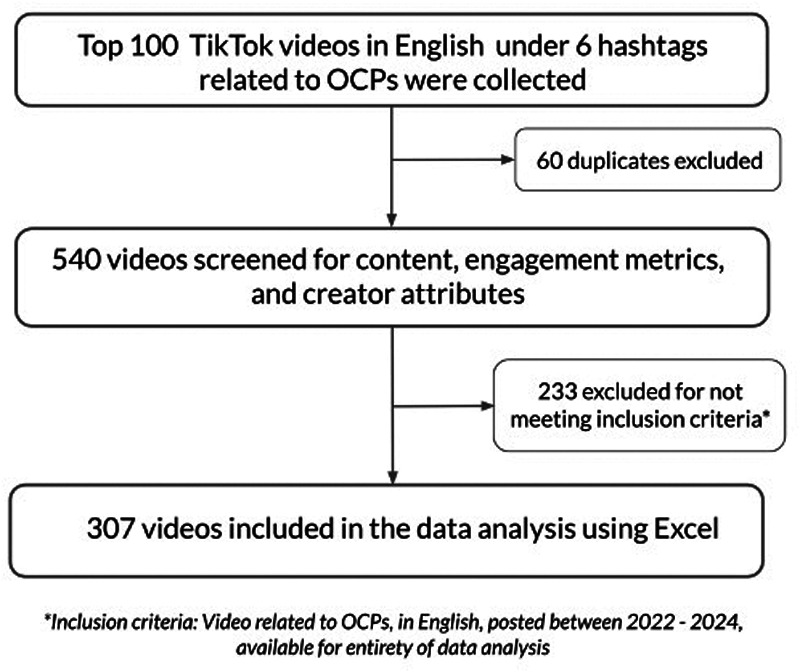
Flow chart of video inclusion methods. Top 100 TikTok videos in English under six hashtags related to oral contraceptive pills (OCPs) were collected. After removing 60 duplicates, 540 videos were screened for content, engagement metrics, and creator attributes. A total of 233 videos were excluded for not meeting inclusion criteria (videos related to OCPs, in English, posted between 2022 and 2024, and available for the entirety of data analysis), resulting in 307 videos included in the final analysis using Excel.

We conducted a quantitative content analysis of TikTok videos related to OCPs. This study was deemed exempt by the Henry Ford Health Institutional Review Board. A new, anonymous TikTok account was created to conduct this study to prevent bias from prior search history or algorithm personalization. Data collection was done on a single day on July 16th, 2024, in which the top 100 TikTok videos in English under each of the following six hashtags related to OCPs were collected: #birthcontrolpill, #birthcontrol, #oralcontraceptive, #birthcontrolproblems, #birthcontrolsideeffects, #thepill. Hashtags were selected based on their direct relevance to OCPs and their high frequency of use observed in prior social media studies on reproductive health. Duplicate videos under multiple hashtags were included only once, which excluded 60 videos. This resulted in 540 unique videos, which were screened for eligibility using the following inclusion criteria: videos must be related to OCPs, in English, posted between 2022 and 2024 (to maintain relevance and feasibility of the study), and available for public viewing for the duration of data analysis. After excluding 233 videos that did not meet the inclusion criteria (168 not related to OCPs, 1 not in English, 57 posted before 2022, and 7 made private during data analysis), a total of 307 videos were included in the final analysis (Figure 1).

The research team created a structured codebook, generating categories based on prior social media content analyses in health communication and themes observed in a pilot review of 30 OCP-related TikTok videos. Codes were refined collaboratively to ensure clarity and applicability. Each of the 307 videos was independently coded by two trained reviewers who analyzed video content, engagement metrics (likes, comments, shares), and creator attributes (number of followers, verification status). Coded content included: user's overall experience description (positive/neutral/negative), tone of video (positive/neutral/negative), video creator (HCP/non-HCP), and scientific citation (yes/no). Non-HCP creators included individual TikTok users, influencers, and organizations without formal healthcare credentials. In addition, we examined the content of the videos for the following: educational (yes/no), comedy (yes/no), logistics of use of the OCP (yes/no), medication mechanism of action (yes/no), discussion of pregnancy (yes/no), side effects (yes/no), accessibility of medication (yes/no), and distrust of medical professionals (yes/no). To illustrate, a video that humorously described personal side effects of OCPs would be coded as: patient experience = negative, tone = negative, creator = non-HCP, citation = no, comedy = yes, side effects = yes.

Codes were primarily binary (yes/no), with patient experience and tone coded using three categories. When videos contained both positive and negative elements, reviewers determined the dominant tone or theme based on the video's overall message and emphasis. Interrater reliability was assessed on the pilot dataset and refined until agreement exceeded acceptable thresholds (>80%). For the main dataset, discrepancies were resolved by a third reviewer. The modified DISCERN scale is an established tool for assessing the reliability of health information in short-form videos based on the following criteria: whether the aims are clear and achieved; whether reliable sources of information are used (e.g., cited publications or a board-certified physician as the speaker); whether the information is balanced and unbiased; whether additional sources are provided for patient reference; and whether areas of uncertainty are acknowledged (15, 16). Based on its criteria, each video was given a score between 0 (low reliability) and 5 (high reliability). All of the data was analyzed using Excel (Microsoft 365, version 16.88). Descriptive statistics were reported as proportions, and chi-squared tests were used to determine statistical significance.

## Results

Of the 600 TikTok videos collected under the six search terms: #birthcontrolpill, #birthcontrol, #oralcontraceptive, #birthcontrolproblems, #birthcontrolsideeffects, and #thepill, 307 unique videos met the inclusion criteria and underwent data analysis. At the time of analysis, the videos had an average of 135,891 likes, 1,080 comments and 7,483 shares. The average number of followers per user was 205,728, and 5.6% (*n* = 17) of the users were verified. Of the 307 videos analyzed, 12.1% (*n* = 37) had a positive tone, 33.6% (*n* = 103) had a neutral tone and 54.4% (*n* = 167) had a negative tone regarding OCPs.

Videos that positively discussed OCPs made claims such as “*Let me tell you about my positive experience on birth control..I feel like I never hear anybody talking about this”* and “*I’m the happiest I’ve ever been…I feel so stable”* (Table 1). Whereas, users who discussed OCPs in a negative tone would make statements such as *“birth control is one of the most damaging things you can put in your body”* and “*If I had a daughter, she would never be allowed to take birth control–I would rather her just get pregnant..all of the long term effects it [birth control] could possibly have on her..yeah no”.*

**Table 1:** Example quotes from TikTok videos assessing positive, neutral, and negative tones.

Example Quotes from TikTok Videos	Tone
"Let me tell you about my positive experience on birth control..I feel like I never hear anybody talking about this"	Positive
"Birth control has changed my life for the better..I've heard so many negative things about it that scares people from going on it, and that just wasn't the case for me"	Positive
“The best birth control is the one that works for you”	Neutral
"I have more energy, my emotions are coming back.. starting to think this [coming off birth control] was the best decision I've ever made"	Negative
"Birth control is one of the most damaging things you can put in your body"	Negative

**Figure 2:**
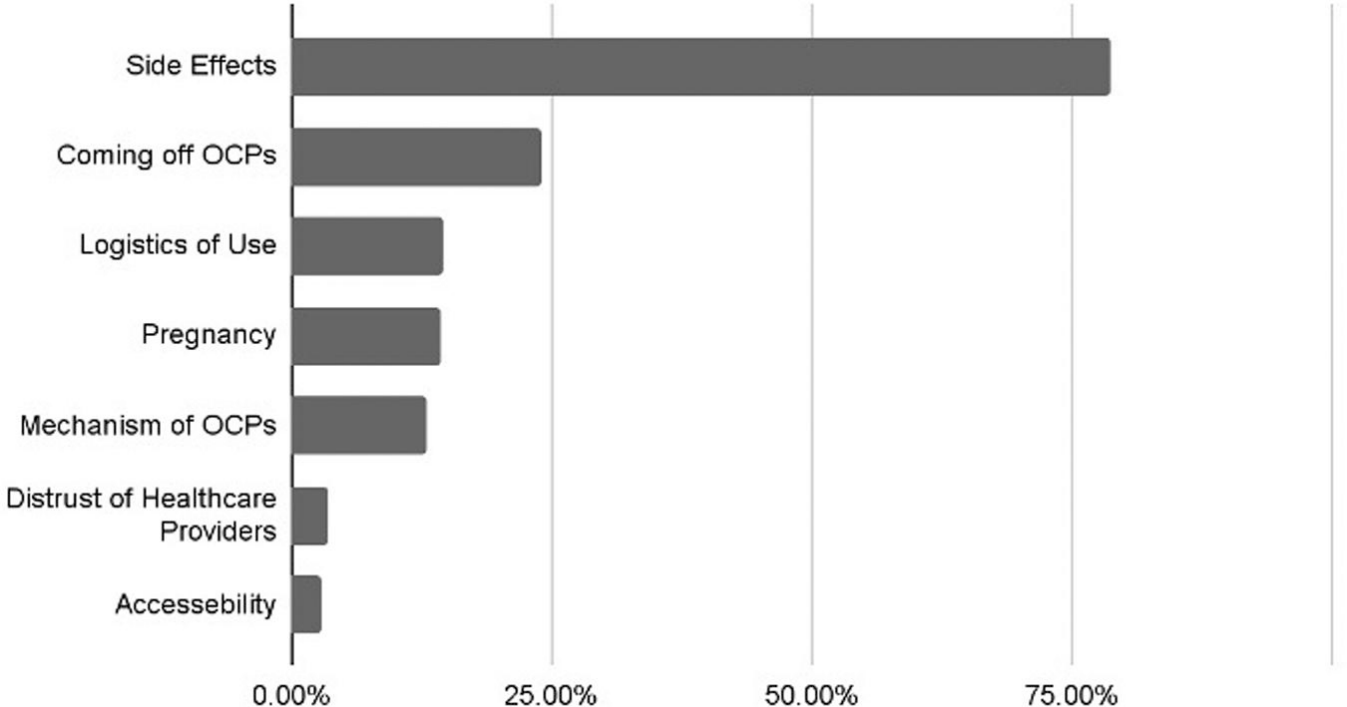
Proportion of TikTok videos discussing each content topic. The most frequently discussed topic was side effects, followed by coming off oral contraceptive pills (OCPs), logistics of using OCPs, pregnancy, and the mechanism of OCPs. Less commonly discussed topics included distrust of healthcare providers and accessibility.

Many videos addressed multiple topics; therefore, each video was coded for all relevant content areas. The most common topic discussed in videos was the side effects of OCPs (78.8%, *n* = 242), with the majority of videos discussing adverse events users experienced while taking the pill (64.0%, *n* = 155/242) (Figure 2). One user said the following statement regarding the side effects experienced on OCPs:

Going on birth control at 15 to “balance my hormones” and fix my period not realizing that it would result in low libido, horrible gut health, frequent UTIs, being viewed as less attractive to men, undiagnosed PCOS, inflammation in my body and lasting puffiness in my face and abdomen.

The second most discussed topic was discontinuation of OCPs (24.0%, *n* = 74), particularly the negative effects of stopping OCPs or the positive experiences users had once they stopped taking the pill (83.8%, *n* = 62/74). One user said the following regarding coming off of OCPs: “*I have more energy, my emotions are coming back..* *starting to think this [coming off birth control] was the best decision I've ever made”.* Another user said the following: “*[being off the pill] it's so worth it physically, mentally, emotionally. I feel so much better than I did when I fit into my smallest pair of jeans.”* Other less commonly discussed topics include logistics of use of the OCP (14.7%, *n* = 45), pregnancy (14.3%, *n* = 44), mechanism of action of OCPs (13.0%, *n* = 40), distrust of HCPs (3.6%, *n* = 11) and accessibility of the medication (2.9%, *n* = 9).

**Figure 3:**
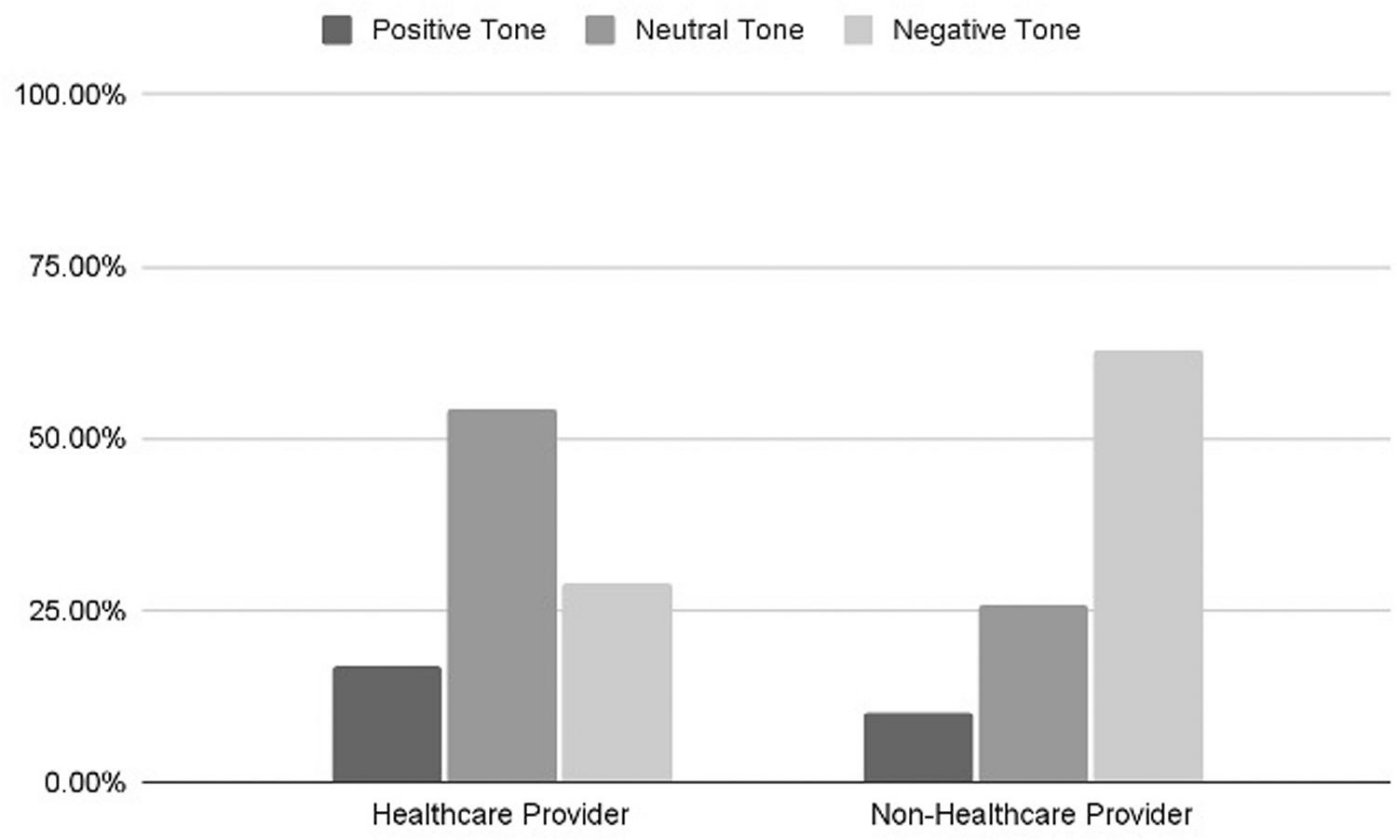
Tone of videos stratified by the type of creator. Videos are classified as positive, neutral, or negative tones created by healthcare providers vs. non-healthcare providers.

The majority of videos analyzed (56.7%, *n* = 174) were coded as testimonials, describing users either sharing personal experiences with OCPs or seeking advice about them. Educational videos were the second most common type, comprising 36.2% (*n* = 111) of the total. Notably, the majority of these educational videos (63.9%, *n* = 71) were created by HCPs. Overall, HCPs were responsible for creating more than a quarter (27.0%, *n* = 83) of all videos, with most of their contributions (85.5%, *n* = 71) being educational. A chi-squared analysis reveals a significant difference in the type of videos created by HCPs and non-HCPs (*p* < 0.001). Of the videos created by HCPs, 16.9% had a positive tone, 54.2% had a neutral tone (*n* = 45), 28.9% had a negative tone (*n* = 24). Comparatively, of the non-HCP videos, 10.0% (*n* = 23) had a positive tone, 25.9% (*n* = 58) had a neutral tone, and 63.0% (*n* = 143) had a negative tone (Figure 3). Utilizing a chi-squared analysis, there is a significant difference in the tone of video comparing HCPs and non-HCPs (*p* < 0.001).

Videos created by HCPs had an average modified DISCERN score of 2.45, demonstrating higher reliability of information compared to videos created by non-HCPs, which scored an average of 0.79 (*p* < 0.05). Of the 307 videos analyzed, only 10 (3.3%) included a scientific citation in the video or caption, 70% of which were created by HCPs. In terms of engagement, videos with a negative tone received significantly more likes than videos with a positive tone, with an average of 187,876 likes for negative and 70,488 for positive videos (*p* < 0.05). Videos created by non-HCPs received significantly more likes than videos by HCPs, with an average of 178,722 likes for non-HCPs and 20,815 likes for HCPs (*p* < 0.05).

## Discussion

This study aimed to analyze the content and reliability of videos discussing OCPs on TikTok. Consistent with prior research, videos with a negative tone received substantially higher engagement than those with a positive tone, reflecting the well-documented negativity bias in online communication (17–19). Some creators who shared positive experiences with OCPs also acknowledged that such narratives are seldom discussed online. This suggests a perceived imbalance in how OCP experiences are represented on TikTok. With the negative narratives dominating public discourse, there is a risk of vilification of OCPs. Repeated exposure to strongly emotive personal experiences may disproportionately shape viewers' perceptions. In the current post-Roe v. Wade era, where access to abortion and comprehensive reproductive healthcare is increasingly limited in many U.S. states, Qato et al. (20) documented declines in oral contraceptive use and emergency contraceptive use. The dominance of negative portrayals of OCPs on TikTok observed in our study may further discourage contraception uptake, with potential implications for unintended pregnancies among adolescents and young adults.

The majority of TikTok videos analyzed in this study were testimonial in nature, with users describing how OCP use affected their mood, weight, or overall well-being and often attributed these changes to the pill. These personal accounts mirror the influence of traditional social networks, where individuals rely heavily on the experiences of family and friends when making contraceptive decisions (2, 3). Our findings suggest that TikTok now functions as an extension of these networks, shaping perceptions of OCPs through shared narratives. It is also important to consider whose stories are represented on TikTok. Users who share reproductive health content are likely to have access to the time and technology required for content creation (21, 22). These digital divides may influence which experiences are amplified and which remain underrepresented. Likewise, the health literacy of both creators and viewers may affect how OCP-related information is interpreted, particularly when personal narratives are presented alongside or instead of evidence-based guidance.

Many creators also shared experiences after discontinuing OCPs, often describing improvements in mood, energy, or physical health and expressing concerns about long-term hormonal exposure or a desire for a more “natural” lifestyle. Pfender et al. (10) observed similar patterns in YouTube videos from 2019 to 2021, suggesting that the interest in discontinuing hormonal contraception in favor of nonhormonal methods is not new. The prevalence of OCP discontinuation narratives is notable given the current context in which access to abortion and comprehensive reproductive healthcare is becoming increasingly restricted in parts of the United States (4). The broader anti-abortion climate, including heightened public rhetoric and shifting cultural attitudes toward reproductive autonomy, may also influence how individuals interpret and share contraception-related experiences online. Although we cannot determine whether the policy environment directly influenced creators’ decisions to stop using OCPs, this context underscores the broader implications of publicly sharing and consuming content that frames discontinuation as desirable. Considering these narratives alongside the current reproductive health climate highlights that the way risks and benefits of OCPs are framed on TikTok may have heightened significance for viewers who are making contraceptive decisions in a more restrictive landscape.

HCPs produced a smaller proportion of videos, and these tended to focus on educational content that scored higher on the DISCERN scale. However, these educational videos garnered significantly less engagement than content created by non-HCPs, which may limit their visibility and impact. Both forms of content can play an important role in how individuals understand and evaluate contraceptive options. However, the current landscape on TikTok is weighted more heavily toward experiential narratives, which may shape viewers' perceptions in the absence of exposure to evidence-informed information. This imbalance highlights an opportunity to explore approaches that bring these perspectives into better alignment on the platform.

### Clinical implications

These findings underscore the importance of discussing contraception openly during clinical encounters, particularly because some creators described discontinuing OCPs without consulting a healthcare provider. While early, patient-centered conversations about discontinuation and alternative methods can support informed decision-making, it is also important to recognize that clinicians themselves may face structural constraints, including limited visit time and competing clinical demands, that affect the depth of contraceptive counseling. Ensuring that patients receive comprehensive information requires attention not only to individual clinician-patient communication but also to the broader organizational and system-level factors that shape these interactions.

Given the low engagement of TikTok videos created by HCPs and the popularity of testimonial experiences on the platform, it may be beneficial for HCPs who create TikTok videos to capitalize on testimonies or build a platform prior to releasing educational videos. An alternative strategy could involve collaborating with non-HCP creators or influencers to deliver evidence-based content in relatable formats, potentially improving reach and engagement among TikTok audiences.

### Research implications

The findings of this study emphasize the need for future exploration of TikTok and OCPs. Future research could examine the extent to which TikTok influences contraceptive decision-making by assessing how content affects users' knowledge, attitudes, and behaviors. In addition, qualitative approaches would provide deeper insight into the complexity of these narratives and help move beyond binary distinctions such as positive vs. negative experiences or HCP- vs. non-HCP–generated content. Future qualitative work could also explore how gender norms (e.g., *“..less attractive to men…”)*, body image pressures (e.g., “*..fit into my smallest pair of jeans”)*, and broader societal expectations influence the way individuals discuss and interpret their contraceptive experiences online. Additionally, longitudinal studies may further help to identify trends in engagement and the evolution of OCP-related narratives over time.

### Strengths and limitations

The strengths of this study include the narrow focus on OCPs, which allowed us to conduct a more specific analysis than previous studies that examined contraception on TikTok. Additionally, the utilization of a standardized procedure to capture content analysis with a high degree of inter-coder reliability ensured consistency, minimizing biases and enabling reproducibility. Though this study had several strengths, there were a few limitations. One limitation includes the translatability of the modified DISCERN scale to assess the reliability of short-form videos, as the scale was initially used for long-form videos. Another limitation of this study is that data were collected at a single time point, which may not capture long-term trends or variations in content over time. Additionally, the research team consisted of medical students, and we acknowledge that our clinical training and healthcare-oriented perspective may have influenced our interpretation of the content.

## Conclusion

TikTok has become an influential source of contraceptive information, and personal narratives that highlight negative experiences often receive the greatest visibility and engagement. These accounts may shape how users perceive OCPs and contribute to an unbalanced online discourse that emphasizes risks or adverse experiences over evidence-based information, a dynamic that holds particular significance in the post-Roe v. Wade era. At the same time, educational content from healthcare professionals remains less prominent on the platform. Collaborative approaches that bring together clinicians and skilled content creators may help bridge this gap by combining clinical accuracy with the relatability that drives online engagement. As reproductive health policy and public discourse continue to evolve, understanding how OCPs are portrayed on social media will be essential for supporting informed contraceptive decision-making.

## Data Availability

The raw data supporting the conclusions of this article will be made available by the authors, without undue reservation.
